# Prevalence of Traumatic Dental Injury in a Tertiary Care Hospital: A Descriptive Cross-sectional Study

**DOI:** 10.31729/jnma.5556

**Published:** 2021-01-31

**Authors:** Snigdha Shubham, Manisha Nepal, Ravish Mishra, Laxmi Kandel, Narayan Gautam

**Affiliations:** 1 Department of Conservative Dentistry and Endodontics, Universal College of Medical Sciences, Bhairahawa, Rupandehi, Nepal; 2 Department of Oral and Maxillofacial Surgery, Universal College of Medical Sciences, Bhairahawa, Rupandehi, Nepal; 3 Department of Biochemistry, Universal College of Medical Sciences, Bhairahawa, Rupandehi, Nepal

**Keywords:** *prevalence*, *retrospective study*, *traumatic dental injury*

## Abstract

**Introduction:**

Traumatic dental injury is an injury inflicted on the dentoalveolar system. It has a physical as well as a psychological impact. Despite this concern, epidemiological data regarding its prevalence is insufficient in the literature of Nepal. Hence, this study's objective was to investigate the prevalence of traumatic dental injuries for the patients visiting Universal College of Medical Sciences, Bhairahawa, Nepal, over five years.

**Methods:**

A descriptive cross-sectional study was conducted using records from the medical record section for the patients presenting at the dental emergency outpatient department of the Universal College of Medical Sciences, Bhairahawa, Nepal, between April 2014 and April 2019. Ethical approval was taken from the Institutional Review Committee of the Universal College of Medical Sciences. Patient demographic data, type of traumatic dental injuries, and etiologies were evaluated from the record section.

**Results:**

Out of 10,080 patients registered during the study period, 793 patients (7.86%) were due to traumatic dental injury, out of which 628 (79.2%) were male, and 165 (20.8%) were female. The most vulnerable age group was 20-29 years (42.4%). Most frequently, injuries occurred in June (16%). Road traffic accidents (57.8%) were the most common mode, and complicated crown-root fracture (23.3%) was the most common type of traumatic dental injury.

**Conclusions:**

The frequency of 7.86% of traumatic dental injury indicates that dental traumatology needs special attention for policy planning and professional training.

## INTRODUCTION

“Traumatic injury” means destruction or damage to the tissues due to trauma, which leads to inflammatory and dystrophic consequences in the afflicted part.^1^ Traumatic dental injury (TDI) is an injury inflicted to the dentoalveolar system.

As the oral cavity is the sixth most frequently injured part of the body, it shows a high impact on the quality of life. They cause physical and psychological concerns leading to negative socialization implications.^2–4^ Hence, TDI must be considered as an imperative dental public health issue.^5^

Population studies about TDI demonstrate a prevalence that ranges from 3.9% to 58.6%.^4^ Despite its high prevalence and negative impacts, there is a lack of literature about Nepal's dental injury. Hence, this study aimed to investigate the prevalence of TDIs of patients presented to the dental emergency service within five years in UCMS.

## METHODS

This descriptive cross-sectional study was designed and implemented using patients’ dental records with TDIs visiting the Department of Conservative Dentistry and Endodontics, Universal College of Medical Sciences, Bhairahawa, Nepal, from April 2014 to April 2019. The study was approved by the Institutional Review Committee (UCMS/IRC/009/20).

The sample size was calculated by,

$$\begin{array}{ll} \text{Sample size (n)} & ={\text{z}}^{\text{2}}\times \text{p(}1-\text{p)}/{\text{e}}^{\text{2}}\\ & =2.6^{2}\times 0.5(1-0.5)/0.02^{\text{2}}\\ & =4225 \end{array}$$

where,

Z = 2.6 at 99% Confidence Interval
p = 50% assumed prevalence of traumatic dental injury
q = 0.5 (1-p)
e = 0.02 at 2% margin of error

The minimum sample size was 4225, but as a non-randomized sampling technique (consecutive sampling) was used, we have doubled the sample size to 8850. So, we have taken a sample of 10,080.

All the cases with isolated dental traumatic injuries who received treatment during the study period were included. Cases with incomplete documentation and associated polytrauma patients were excluded from the study. The following information was collected from each patient's record: gender, age at the time of injury, the month of trauma, cause of injury, and type of dental injury. The causes of TDI were classified into six categories and coded as 1) Fall, 2) Road traffic accidents, 3) Sports activities, 4) Collisions, 5) Physical assaults 6) Others. The type of TDIs was recorded, according to the system described by Andreasen and Andreasen^6^ as 1) Crown infraction 2) Uncomplicated crown fracture 3) Complicated crown fracture 4) Uncomplicated crown-root fracture 5) Complicated crown-root fracture 6) Root fracture 7) Concussion 8) Subluxation 9) Lateral luxation 10) Extrusive luxation 11) Intrusive luxation 12) Exarticulation (avulsion).

The data were entered in Microsoft Excel. The statistical analyses were performed using Statistical Package for Social Sciences (SPSS) software program for Windows 21.0 statistical software (SPSS Inc., Chicago, IL, USA) at a point estimate of 99% confidence interval. Qualitative data are presented as frequencies and percentages.

## RESULTS

Out of the 10,080 patients seen in the five years, 793 (7.86%) patients presented with TDIs. Among which more men, 628 (79.2%) presented with TDI than females 165 (20.8%) (Table 1).

**Table 1:** Distribution of TDI based on gender.

Gender	n (%)
Male	628 (79.2)
Female	165 (20.8)
Total	793 (100)

A maximum number of traumatic dental injuries occurred in the age group of 20-29 (42.4%), followed by 30-39 (14.9%) and 40-49 (14.5%) age group, respectively (Table 2).

**Table 2:** Distribution of TDI according to age-group.

Age-group	n (%)
0-9	42 (5.3)
10-19	108 (13.6)
20-29	336 (42.4)
30-39	118 (14.9)
40-49	115 (14.5)
50-59	36 (4.5)
60-69	28 (3.5)
70-79	10 (1.3)
Total	793 (100)

Concerning the month of the visit of trauma cases, June was the month with the highest number, 127 (16%) of trauma, followed by February 98 (12.4%)(Table 3).

**Table 3:** Distribution of TDI according to month.

Month	n (%)
January	62 (7.8)
February	98 (12.4)
March	54 (6.8)
April	57 (7.2)
May	34 (4.3)
June	127 (16)
July	70 (8.8)
August	66 (8.3)
September	23 (2.9)
October	54 (6.8)
November	51 (6.4)
December	97 (12.2)
Total	793 (100)

The most frequent reasons for TDIs were road traffic accidents 458 (57.8%), followed by fall injury 112 (14.1%) and physical assaults 100 (12.6%) (Table 4).

**Table 4:** Distribution of TDI according to modes of trauma.

Modes of trauma	n (%)
Fall	112 (14.1)
Road traffic accidents	458 (57.8)
Physical assault	100 (12.6)
Sports accidents	69 (8.7)
Collision	31 (3.9)
Others	23 (2.9)
Total	793 (100)

The complicated crown-root fracture was the most common type of TDIs 185 (23.3%), followed by avulsion 103 (13%) (Table 5).

**Table 5:** Distribution of TDI according to type.

Type of TDI	n (%)
Crown fracture	64 (8.1)
Uncomplicated crown fracture	34 (4.3)
Complicated crown fracture	47 (5.9)
Uncomplicated crown root fracture	15 (1.9)
Complicated crown root fracture	185 (23.3)
Root fracture	64 (8.1)
Concussion	67 (8.4)
Subluxation	43 (5.4)
Lateral luxation	73 (9.2)
Extrusive luxation	56 (7.1)
Intrusive luxation	42 (5.3)
Avulsion	103 (13)
Total	793 (100)

## DISCUSSION

Epidemiological data of TDIs is important for the population of Nepal as the literature lacks ample data. The findings can help develop preventive strategies and the formulation of definite clinical decisions with remedial protocols.^5^ Hence, the present study was done to determine the prevalence of TDIs in the trauma patients visiting to seek medical and surgical care at the tertiary care center of Rupandehi, Nepal.

The prevalence of dental trauma is 7.86% in the present study, whereas prevalence is 4.15% in India,^7^ 27.7% in the United Kingdom, 11% in Greece, and 8.4% in France.^8^ The difference in prevalence might be due to socioeconomic, behavioral, cultural diversities, and geographical variations of the study's locations.

TDIs classification recorded in the present study was based on Andreasen and Andreasen's criteria, which is similar to the studies done previously.^8–9^ This classification is based on anatomic, therapeutic, and prognostic considerations which better predicts the severity and state of TDI.^5^

The present study showed a greater number of men presented with TDI than women; as men are more likely to be outgoing, they participate in more aggressive types of sports and adventurous driving. The result is in accordance with the study done by Mahmoodi et al.,^8^ Shrestha et al.^9^ and Adhikari et al.^10^ Age group of 20-29 had maximum TDIs which might be due to rough driving of motorbikes on the road, road traffic accidents under the influence of alcohol, negligence in using safety measures while riding such as helmets or any other protective covers and involvement in a more aggressive type of sports. Besides, this age group is more productive because they are more exposed to the outside environment and undergo work-related stress. The result contrasts with a study done by Borin-Moura et al.^11^, which shows that the 13-19 years of age group has maximum TDI cases.

The month-wise distribution showed that the maximum number of cases occurred in June (16%), which contrasts with the study done by Guedes OA et al.^4^, which showed maximum TDIs in the month of July-September. More cases of TDI in June in the present study might be due to the slippery road in monsoon season; moreover, Nepal's roads are in poor condition in the rainy season.

The most common cause of TDIs was a road traffic accident (57.8%) because Nepal is a developing country with dangerous landscapes and poor road conditions. Also, pedestrians have no seclusion from wheeled traffic, neglect of traffic rules, and substandard design of crossroads and speed breakers.

The most common injury in our investigation was complicated crown-root-fracture (23.3%). In contrast, a study conducted by Shrestha et al.^9^ and Borin-Moura et al.^11^ showed an uncomplicated crown fracture to be more prevalent, which accounts for 44.4% and 52.6%, respectively.

This study gives an insight into the magnitude of different types of traumatic dental injuries in a particular region of Nepal (i.e., Rupandehi). Hence, a multicentric, nation-wide survey is required to understand better the degree of severity, which will help policy formulation. This study can contribute to some extent to an idea of the epidemiology of the TDIs even though it is a hospital-based study. The findings can help develop preventive guidelines and the adoption of precise remedial judgments with therapeutic protocols. Future prospective studies based on patients’ follow-up are needed to evaluate the therapeutic protocols’ efficacy and their implications over time.^5^

## CONCLUSIONS

The prevalence of TDI is 7.86% in this study, which is only the tip of the iceberg because the major injuries beyond the scope of one tertiary care center were not included in this study. The study is conducted to assess the magnitude of the injury, which can further contribute to quantifying the burden of TDI. Policy and guidelines need to be formulated and implemented by the responsible sectors to prevent and manage it. Road and motor vehicle conditions should be monitored regularly and promptly as it is directly accountable for accident occurrence.
